# Impact of Methyl Jasmonate on Terpenoid Biosynthesis and Functional Analysis of Sesquiterpene Synthesis Genes in *Schizonepeta tenuifolia*

**DOI:** 10.3390/plants13141920

**Published:** 2024-07-12

**Authors:** Dishuai Li, Congling Jia, Guyin Lin, Jingjie Dang, Chanchan Liu, Qinan Wu

**Affiliations:** 1 Jiangsu Collaborative Innovation Center of Chinese Medicinal Resources Industrialization, Nanjing University of Chinese Medicine, Nanjing 210023, China; 20210651@njucm.edu.cn (D.L.); jiacongling@njucm.edu.cn (C.J.); lgy_grace@sina.com (G.L.); jingjiedang@njucm.edu.cn (J.D.); 2 State Key Laboratory on Technologies for Chinese Medicine Pharmaceutical Process Control and Intelligent Manufacture, Nanjing University of Chinese Medicine, Nanjing 210023, China; 3 School of Pharmacy, Nanjing University of Chinese Medicine, Nanjing 210023, China

**Keywords:** methyl jasmonate, *Schizonepeta tenuifolia*, germacrene D synthase, terpenoid component, adventitious bud clusters

## Abstract

This study investigates the impact of methyl jasmonate (MeJA) on the volatile oil composition of *Schizonepeta tenuifolia* and elucidates the function of the *StTPS45* gene, a key player in terpenoid biosynthesis. The effect of different concentrations of MeJA (0, 50, 100, 200, and 300 μmol/L) on the growth of *S. tenuifolia* adventitious bud clusters was analyzed over a 20 d period. Using gas chromatography–mass spectrometry (GC-MS), 17 compounds were identified from the adventitious bud clusters of *S. tenuifolia*. Significant changes in the levels of major monoterpenes, including increased contents of (+)-limonene and (+)-menthone, were observed, particularly at higher concentrations of MeJA. Analysis of transcriptome data from three groups treated with 0, 100, and 300 μmol/L MeJA revealed significant changes in the gene expression profiles following MeJA treatment. At 100 μmol/L MeJA, most terpene synthase (TPS) genes were overexpressed. Additionally, gene expression and functional predictions suggested that *StTPS45* acts as germacrene D synthase. Therefore, *StTPS45* was cloned and expressed in *Escherichia coli*, and enzyme activity assays confirmed its function as a germacrene D synthase. Molecular docking and structural prediction of *StTPS45* further suggested specific interactions with farnesyl diphosphate (FPP), aligning with its role in the terpenoid synthesis pathway. These findings provide valuable insights into the modulation of secondary metabolite pathways by jasmonate signaling and underscore the potential of genetic engineering approaches to enhance the production of specific terpenoids in medicinal plants.

## 1. Introduction

*Schizonepeta tenuifolia*, commonly known as Japanese catnip, is a valuable medicinal plant in the Lamiaceae family, renowned for its antipyretic, anti-inflammatory, and analgesic properties in traditional Chinese medicine [1]. The therapeutic effects of *S. tenuifolia* are largely attributed to its rich volatile oil content, which includes significant terpenoids such as (−)-pulegone, (+)-limonene, (−)-isopulegone and (+)-menthone [2]. These bioactive compounds exhibit diverse pharmacological activities: (−)-pulegone possesses anti-hyperalgesic properties [3]; (+)-limonene is known for its anti-anxiety and anticancer effects [4,5]; (−)-isopulegone has antimicrobial activity [6]; and (+)-menthone is valued for its anti-allergic and antitumor properties [7,8]. These attributes make them the focus of extensive research into their biosynthesis and regulation.

**Figure 1:**
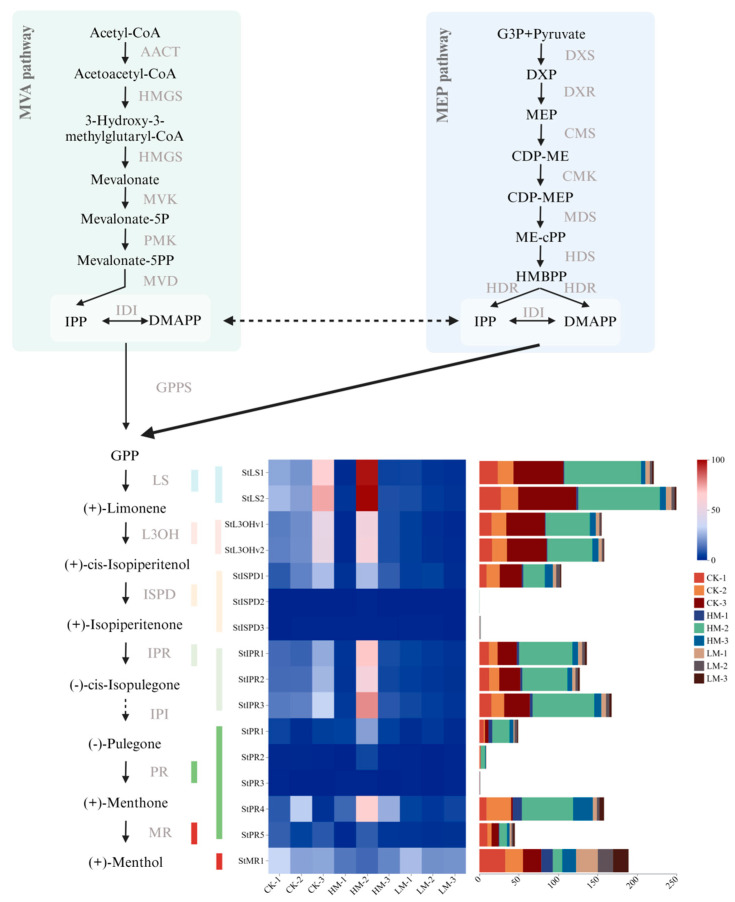
The synthesis pathway of (+)-menthol in *S. tenuifolia* and the gene expression levels of key enzymes involved in this pathway.

The synthesis pathway of monoterpenes in *S. tenuifolia* begins with the MEP and MVA pathways, which together produce isopentenyl pyrophosphate (IPP) and dimethylallyl pyrophosphate (DMAPP) [9,10]. These compounds then undergo a series of reactions catalyzed by terpene synthases (TPS), including those catalyzed by limonene synthase (LS), limonene-3-hydroxylase (L3OH), isopiperitenol dehydrogenase (ISPD), isopiperitenone reductase (IPR), pulegone reductase (PR), and menthone reductase (MR), ultimately resulting in the synthesis of (+)-menthol (Figure 1). The genes encoding these enzymes form unique bidirectional gene clusters in the *S. tenuifolia* genome, indicating that these genes are grouped together through inverted duplication, and work synergistically to accomplish the biosynthesis of monoterpenoids [11]. Additionally, analyses of the *TPS* gene family members in *S. tenuifolia*, including their physicochemical properties, phylogenetic relationships, conserved motifs, and gene structures, have also been reported [12]. However, there has been no progress in the research on the sesquiterpene synthesis pathway in *S. tenuifolia*.

Previous studies have shown that terpenoid compounds are influenced by various factors, including light [13], salt stress [14], temperature [15], and endogenous plant hormones [16,17]. Methyl jasmonate (MeJA), an endogenous plant hormone, plays a crucial role in regulating the biosynthesis of secondary metabolites, including terpenoids [18]. Studies have demonstrated that 0.1 mM MeJA can effectively promote the biosynthesis of terpenoids in *Oenanthe javanica* [19]. MeJA can induce the production of terpenoids by activating specific genes involved in their biosynthetic pathways [20,21]. However, the precise mechanisms by which MeJA influences terpenoid biosynthesis in *S. tenuifolia* have not been fully elucidated.

This study used clonal adventitious bud clusters for MeJA induction experiments because they offer greater uniformity and controllability in genetic background, physiological state, and experimental conditions, thereby enhancing the precision and reproducibility of the experiments. Different concentrations of MeJA were applied to the adventitious bud clusters of *S. tenuifolia*, and changes in growth, volatile oil composition and content, and gene expression levels were recorded to elucidate the regulatory role of jasmonate signaling in terpenoid biosynthesis. Additionally, the germacrene D synthase gene *StTPS45* was cloned, expressed, and functionally characterized to confirm its role. Enzyme activity assays and molecular docking studies were conducted to further understand the biochemical properties and interaction mechanisms between *StTPS45* and its substrate, farnesyl diphosphate (FPP). These findings will provide a foundation for optimizing the production of valuable medicinal compounds in *S. tenuifolia* and other related medicinal plants.

## 2. Methods and Materials

### 2.1. Plant Material

This study utilized plant materials from a laboratory setting, which were identified by professor Qinan Wu from the school of pharmacy at Nanjing University of Chinese Medicine as *Schizonepeta tenuifolia*, a species within the Lamiaceae family. *S*. *tenuifolia* seeds were disinfected with 75% ethanol for 2 min, followed by 20% hydrogen peroxide for 6 to 8 min, then rinsed 6 to 8 times with sterile water, and sown on MS culture medium. The seeds were germinated under aseptic conditions in an incubator maintained at 25 °C with a 16 h photoperiod. After approximately 5 days (d), the germinated seeds were cultivated for over 30 d to obtain healthy, sterile seedlings of *S*. *tenuifolia*. Sterile *S*. *tenuifolia* seedlings grown for 60 d were removed from the culture bottles, and stem segments with leaf axils were cut and inoculated onto MS differentiation medium. Under conditions of 25 °C and a light intensity of 2200 lux with a 16 h photoperiod, the seedlings were cultured for 30 d to induce bud generation through methyl jasmonate (MeJA) treatment. Fresh leaves were first immersed in pure water for cleaning, then dried with filter paper to prepare for total RNA extraction.

### 2.2. Effects of MeJA Treatment on Volatile Components of Schizonepeta tenuifolia

#### 2.2.1. MeJA Induces the Formation of *Schizonepeta tenuifolia*

Healthy, uniformly grown, and non-browned *S. tenuifolia* adventitious bud clusters, pre-cultivated for 30 d, were inoculated onto MS culture medium containing 0 (CK), 50 (TM), 100 (LM), 200 (MM) and 300 (HM) μmol/L MeJA. Three bottles were prepared for each concentration, with each bottle containing five samples. The experiment was replicated three times. These groups were cultivated at 25 °C with 70–80% humidity and sampled at 0, 5, 10, 15, and 20 d. Samples were preserved at −80 °C for subsequent uniform extraction and analysis of relevant indicators.

#### 2.2.2. Effect of MeJA Treatment on the Growth of *Schizonepeta tenuifolia*

The proliferation rate of *S. tenuifolia* adventitious bud clusters was calculated using the following formula: Proliferation Rate (%) = [(Weight at Harvest − Weight at Inoculation)/Weight at Inoculation] × 100%. Observations and recordings of the glandular scale phenotype on the leaves of the adventitious buds were conducted using a Zeiss stereomicroscope. For each concentration, three plants were randomly selected for observation. The diameter of six glandular scales was measured using the length measurement tool in the microscopy photography software (ZEN Blue Edition v3.8). Additionally, the number of glandular scales was counted within three known area regions using the area measurement tool, and their density (number/mm^2^) was calculated. The average of these measurements was then determined.

#### 2.2.3. Effect of MeJA Treatment on Volatile Components of *Schizonepeta tenuifolia*

Volatile oils were extracted from MeJA-induced *S. tenuifolia* sterile adventitious bud clusters using simultaneous distillation extraction. The extraction involved 8 mL of n-hexane for 4 g of fresh bud tissue over a period of 1 h. The extracted samples were then analyzed for content using gas chromatography–mass spectrometry (GC-MS). The GC-MS was equipped with an Agilent 19091S-433 HP-5ms column (30 m × 250 μm × 0.25 μm), and helium was used as the carrier gas. The injector temperature was set at 250 °C. The temperature program started at 50 °C, held for 32 min, then increased at 10 °C/min to 90 °C and held for 15 min, followed by an increase at 5 °C/min to 200 °C and held for 5 min. The split ratio was 20:1, and the injection volume was 1 μL. Mass spectrometry conditions included an electron ionization (EI) source at 70 eV, source temperature of 230 °C, quadrupole temperature of 150 °C, and a mass scan range of 30~500 amu, with a solvent delay of 3 min. Component identification was conducted by comparing retention times and mass spectral data with standards and using the NIST database. Total ion chromatograms were obtained and further analyzed against the NTST.14 database (https://www.nist.gov/) to identify the chemical components in the volatile oil of *S. tenuifolia*.

### 2.3. RNA Extraction, cDNA Library Construction and Data Assembly

Total RNA was extracted from *S. tenuifolia* leaves treated with CK, LM, and HM using TRIzol reagent (Sigma, St. Louis, MI, USA). The mRNA containing poly-A tails was enriched using oligo-dT magnetic beads. A DNA probe hybridized with rRNA, and RNase H selectively digested the DNA/RNA hybrid strand. DNase I was then used to remove the DNA probe, yielding the purified RNA. RNA was fragmented with a breakage buffer and reverse-transcribed using random N6 primers to synthesize double-stranded cDNA. The synthesized double-stranded DNA was end-repaired and 5′-phosphorylated to produce 3′-overhanging “A” sticky ends. A bubble adapter with a 3′-overhanging “T” was then ligated to the DNA. The ligated DNA was PCR-amplified using specific primers, heat-denatured to form single-stranded DNA, and circularized with a bridge primer to create a single-stranded circular DNA library. The library was then sequenced. Data were processed using SOAPnuke (v1.4.0) [22] and Trimmomatic (v0.36) [23] to filter out low-quality reads, adapter contamination, and reads with a high N base content. The clean reads were aligned to the reference genome using HISAT (v2.1.0) [24], and gene expression levels were calculated with StringTie (v2.2.3) [25], using fragments per kilobase of transcript per million mapped reads (FPKM) as the standard measure. PCA and Pearson’s correlation analysis of gene expression values (FPKM) were conducted to evaluate the differences between groups. Differentially expressed genes were identified with DESeq2 [26], using |log_2_(FoldChange)| > 1 and FDR < 0.05 as selection criteria. Volcano plots were used to visualize gene expression differences. Functional and pathway analyses were then conducted using GO [27] and KEGG [28] to reveal the functions and signaling pathways involved.

### 2.4. Optimization and Prokaryotic Expression of Candidate Gene of Germacrene D Synthase

To enhance the expression of TPS45 protein in *E. coli*, a 1695 bp DNA sequence was optimized (Figure S1). The optimized sequence was synthesized by Sangon Biotech (Shanghai) Co., Ltd. (Shanghai, China), and ligated into the pET32a vector to construct the recombinant plasmid pET32a-*TPS45*. This plasmid was transformed into BL21 (DE3) competent cells and inoculated using a positive single colony into 5 mL of LB medium containing 100 ng/mL ampicillin. The culture was then scaled up by inoculating 1 mL of the recombinant culture into 50 mL of ampicillin-containing LB medium and incubated at 37 °C with shaking at 180 rpm until the OD600 reached 0.6–0.8. Isopropyl *β*-D-1-thiogalactopyranoside (IPTG) was added to a final concentration of 1 mmol/L to induce protein expression. The induced culture was expressed at 16 °C and 180 rpm for 18 h, with a control culture set up simultaneously. Post-induction, the culture was transferred to 50 mL sterile centrifuge tubes and centrifuged at 5000 rpm for 15 min at 4 °C to precipitate the bacteria. The supernatant was discarded, and the precipitate was resuspended in 1 mL of lysis buffer. The resuspended culture was sonicated for 5 min in an ice bath at 4 °C and centrifuged under the same conditions. The supernatant was transferred to a new 2 mL centrifuge tube, and the precipitate was resuspended in 1 mL of lysis buffer and stored at −80 °C in another 2 mL centrifuge tube.

### 2.5. Functional Validation of the Protein Encoded by Candidate Gene

To verify the function of the TPS45 target protein, substrate feeding was employed [19]. All procedures were performed on ice. A total volume of 500 μL of reagents was sequentially added to 2 mL sample vials (Table S2), with protein expressed from an empty vector serving as the negative control.

After adding the reagents, 200 μL of n-hexane was immediately added to seal the liquids. The vial cap was tightened and sealed with film. The reaction was conducted at 31 °C for 16 h. Afterward, the reaction mixture was stored at −80 °C for 1 h to freeze. Then, using a sampler, the organic layer was carefully extracted and transferred to a sample vial lined with an inner tube, where it was temporarily stored at −80 °C.

### 2.6. Analysis of the Enzyme Products of the Candidate Gene by GC-MS

The analysis of relevant components was conducted using a GC-MS system (Agilent 7890B-7000D) (Agilent Technologies, Santa Clara, CA, USA) equipped with an HP-5ms capillary column (30 m × 250 μm × 0.25 μm, Agilent 19091S-433) (Agilent Technologies, Santa Clara, CA, USA). The temperature program for TPS45 enzyme activity detection was as follows: the initial temperature was held at 50 °C for 3 min, then increased at a rate of 3 °C/min to 90 °C, followed by 5 °C/min to 150 °C, and finally at 10 °C/min to 220 °C, where it was held for 5 min. Mass spectrometry conditions were as follows: EI mode at 70 eV, ion source temperature of 230 °C, and a mass range of 50–500 amu with an acquisition frequency of 50 Hz. Data collection and analysis were performed using ChemStation software vC.01.05 (Agilent Technologies, Santa Clara, CA, USA), and compounds were identified by comparing mass spectral data against the NIST14.L database and further verified using standards.

### 2.7. Structural Prediction and Molecular Docking of the Protein Encoded by the Candidate Gene

This study conducted a physicochemical analysis of the functionally validated TPS45 protein and predicted its secondary and tertiary structures. Additionally, molecular docking was performed to predict interactions between the TPS45 protein and the FPP small molecule ligand, aiming to identify active sites and infer mechanisms of action. Initially, physicochemical properties were analyzed using various tools: signal peptide prediction with Signal P 4.1 [29] (https://services.healthtech.dtu.dk/service.php?SignalP-4.1, accessed on 10 July 2023), transmembrane regions predicted by TMHMM2.0 (https://services.healthtech.dtu.dk/services/TMHMM-2.0/, accessed on 10 July 2023), glycosylation sites using NetNGlyc 1.0 Server [30] (https://services.healthtech.dtu.dk/service.php?NetNGlyc-1.0, accessed on 10 July 2023), and kinase phosphorylation sites with NetPhos 3.1 Server [31] (https://services.healthtech.dtu.dk/service.php?NetPhos-3.1, accessed on 15 July 2023). Secondary structure was analyzed using SOPMA (https://npsa-prabi.ibcp.fr/cgi-bin/npsa_automat.pl?page=npsa_sopma.html, accessed on 15 July 2023), while three-dimensional modeling was performed via SWISS-MODEL [32] (https://swissmodel.expasy.org/interactive, accessed on 15 July 2023), and the model’s validity assessed with a Ramachandran Plot. Additionally, receptor preparation steps included dehydration and complete hydrogenation. FPP’s chemical structure was prepared using MGLTools (v1.5.6) (https://ccsb.scripps.edu/mgltools, accessed on 22 July 2023) for hydrogen addition and charge calculations. The format was converted from PDBQT to PDB using OpenBabel (v3.0.1) (http://openbabel.org/wiki/Category:Installation, accessed on 23 July 2023), followed by molecular docking with AutoDock [33] (https://autodock.scripps.edu, accessed on 25 July 2023). Interaction analysis between the protein and ligand was visualized and assessed using Discovery Studio 2022 Client (www.imatsoft.com). Finally, amino acid sequence alignments were performed using Clustal Omega [34] (https://www.ebi.ac.uk/Tools/msa/clustalo, accessed on 26 July 2023), and a phylogenetic tree was constructed using the neighbor-joining method in MEGA11 v11.0.10 [35] software.

## 3. Result

### 3.1. Effect of MeJA Treatment on the Phenotype of Schizonepeta tenuifolia

**Figure 2:**
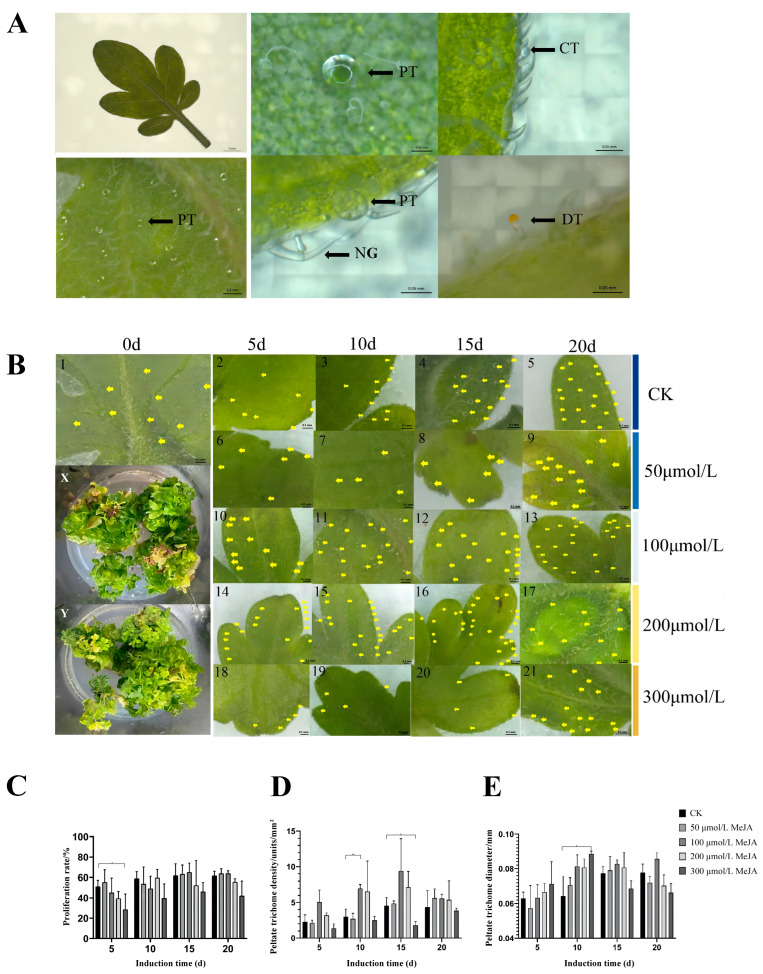
Morphological and physiological responses of *S. tenuifolia* to MeJA treatment. (**A**) Microscopic characteristics of sterile seedlings in tissue culture of *S. tenuifolia.* PT: glandular scale; CT: capitate trichome; DT: digitiform trichome; NG: non-glandular hairs. (**B**) The distribution of glandular scales on the leaves of adventitious buds treated with different concentrations of MeJA. In the X group, clustered adventitious buds were treated with 100 μmol/L MeJA for 15 d, while the Y group served as the CK and was cultured for 15 d. Leaf samples 1 to 5 represent the CK cultured for different time periods. Samples 6 to 9 were treated with 50 μmol/L MeJA for varying durations, 10 to 13 were treated with 100 μmol/L MeJA, 14 to 17 with 200 μmol/L MeJA, and 18 to 21 with 300 μmol/L MeJA, each for different lengths of time. The scale bar is uniformly 0.2 mm, and all observations were made on the abaxial surface of the leaves. Yellow arrows indicate glandular scales. (**C**) The changes in the proliferation rate of *S. tenuifolia* clustered adventitious buds under MeJA treatment. (**D**) The changes in the glandular scale density of *S. tenuifolia* under MeJA treatment. (**E**) The changes in the diameter of *S. tenuifolia* glandular scale under MeJA treatment. ** *p* < 0.01, * *p* < 0.05.

The stereomicroscope revealed three types of glandular hairs on the leaves and stems of sterile *S. tenuifolia* seedlings: glandular scales, digitiform trichomes, and capitate trichomes (Figure 2A). The morphology and structure of glandular hairs in the leaves of sterile tissue-cultured *S. tenuifolia* seedlings were normal, resembling those of known cultivated *S. tenuifolia*. Stem segments with leaf axils from tissue-cultured seedlings at around 30 d, during their vigorous growth stage, were used as explant samples for subsequent MeJA induction experiments.

Treatment of *S. tenuifolia* adventitious bud clusters with different concentrations of MeJA for various durations showed a slight impact on proliferation (Figure 2C). Phenotypic observations revealed that higher concentrations of MeJA resulted in more severe browning at the base of the buds, leading to some loss of plant samples. Specifically, a significant reduction in proliferation rate was observed in the HM group for 5 d (0.01 < *p* < 0.05). However, the LM and MM groups for 15–20 d showed a trend of increased proliferation compared to the CK group, suggesting that moderate concentrations of MeJA might promote the growth and development of *S. tenuifolia* adventitious buds. In contrast, high concentrations of MeJA may adversely affect the normal development of the buds, leading to decreased proliferation rates.

Different concentrations of MeJA variably affect the distribution, diameter, and density of glandular scales on the abaxial side of *S. tenuifolia* leaves (Figure 2B,D,E). In the CK, glandular scales were initially sparsely distributed, but their number and density increased over time, predominantly at the distal edges of the leaves, with fewer glandular scales near the proximal ends. In the TM group, the glandular scale distribution and density resembled that of the CK group, indicating that this lower concentration had no significant effect. However, the LM and MM groups resulted in a marked increase in glandular scale density compared to CK, with glandular scales more widely distributed across the entire leaf surface and particularly denser at the distal edges. Additionally, the diameter of glandular scales also increased to varying degrees. In the HM group, there was a significant decline in glandular scale density from 5 to 15 d, with reductions observed at both leaf ends and almost no glandular scales at the proximal ends. After 20 d at this concentration, glandular scale density slightly increased, returning to a distribution pattern similar to normal, mainly at the distal ends. This recovery could be due to a decrease in MeJA concentration within the plant tissues over time.

### 3.2. Effects of MeJA Induction on Main Terpenoids in Volatile Oil from Schizonepeta tenuifolia

**Figure 3:**
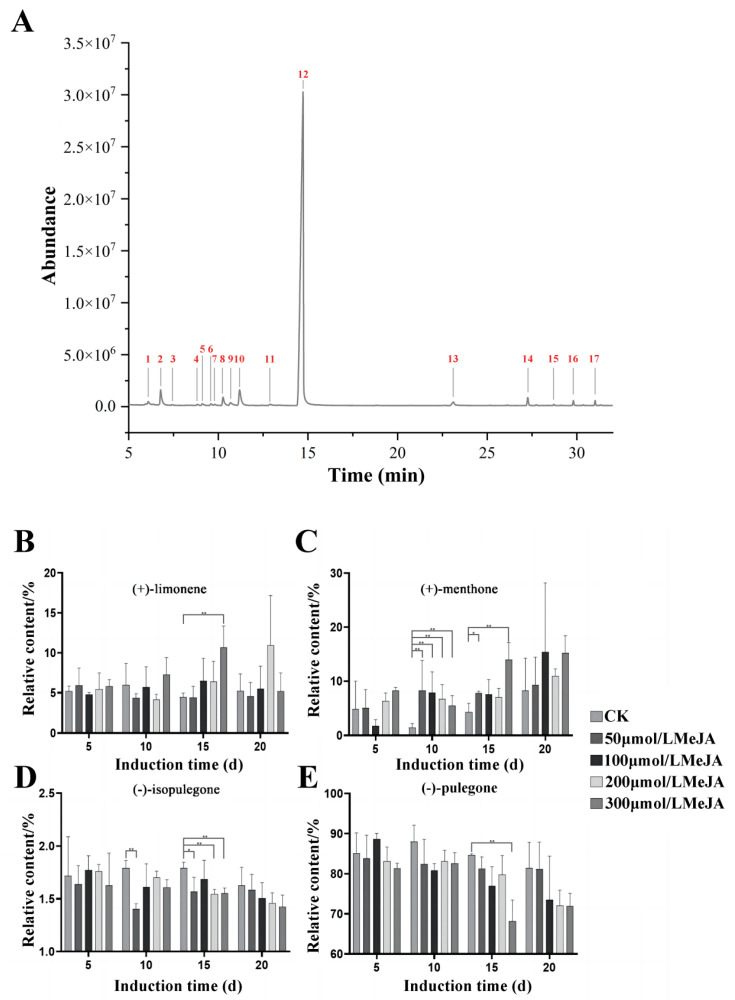
Effects of MeJA on monoterpene components in tissue-cultured *S. tenuifolia* and chemical characterization. (**A**) The GC-MS total ion chromatogram of tissue-cultured *S. tenuifolia* leaves. Effects of different concentrations and induction durations of MeJA on (+)-limonene (**B**), (+)-menthone (**C**), (−)-isopulegone (**D**), and (−)-pulegone (**E**). ** *p* < 0.01, * *p* < 0.05.

The CK group (untreated with MeJA) samples of tissue-cultured *S. tenuifolia* clustered adventitious buds were extracted using simultaneous distillation extraction and analyzed via GC-MS to obtain a total ion chromatogram (Figure 3A). The results were compared with the NIST.14 library and existing standards, summarizing compounds with a similarity score greater than 90% (Table 1). Seventeen distinct compounds were identified, and the compound composition aligned with prior studies [2]. This indicates that the primary monoterpene components in tissue-cultured *S. tenuifolia* essential oil are similar to those in the wild type. The main components remain (+)-limonene, (+)-menthone, (−)-isopulegone, and (−)-pulegone, which together account for about 90% of the total volatile oil in tissue-cultured *S. tenuifolia* clustered adventitious buds.

Adventitious bud clusters of *S. tenuifolia* were induced with 0, 50, 100, 200, and 300 μmol/L concentrations of MeJA for 5, 10, 15, and 20 d. Following induction, volatile oils were extracted using simultaneous distillation extraction and analyzed by GC-MS. The relative concentrations of chemical components were quantified using peak area normalization. The accumulation patterns of the main monoterpenes, particularly menthane derivatives in *S. tenuifolia*, were observed at different exogenous MeJA concentrations and durations (Figure 3B–E). The content of (+)-limonene did not change significantly during the induction process. However, after 15 to 20 d of induction at LM and MM groups, an upward trend in (+)-limonene content was noted. At HM group, a significant increase in (+)-limonene was observed from 5 to 15 d, which then stabilized by 20 d, suggesting that high concentrations of MeJA might positively regulate the synthesis and accumulation of (+)-limonene. In contrast, the content of (+)-menthone increased significantly with both the concentration and duration of induction compared to the CK, with noticeable increases across all concentrations at 20 d. Similarly, the accumulation patterns of (−)-isopulegone and (−)-pulegone showed a gradual decrease with increasing concentrations and duration of induction, with a more pronounced reduction in (−)-pulegone content, indicating that exogenous MeJA might negatively regulate the accumulation of these components, possibly by promoting their conversion into downstream (+)-menthone.

### 3.3. Transcriptome Analysis and Screening of Candidate Genes for Germacrene D Synthase

**Figure 4:**
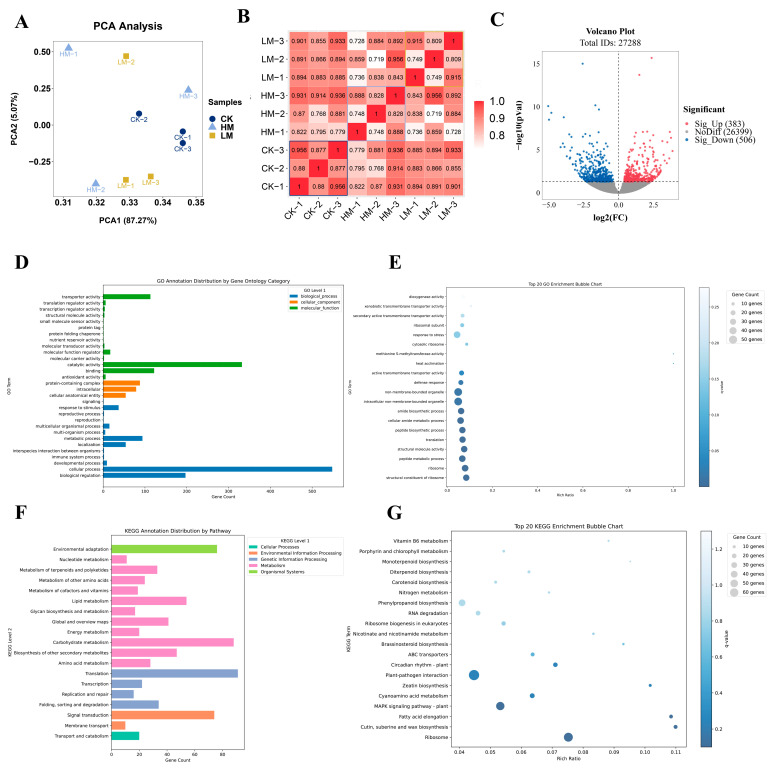
Comprehensive analysis of gene expression in response to MeJA treatment. (**A**) PCA analysis of different treatment groups. (**B**) Pearson’s correlation heatmap of treatment groups. (**C**) Volcano plot of differentially expressed genes in HM compared to CK. (**D**) GO enrichment analysis of differentially expressed genes in HM and CK groups. (**E**) Analysis of top 20 differential GO terms between CK and HM based on q-values. (**F**) KEGG enrichment analysis of differentially expressed genes in HM and CK groups. (**G**) Analysis of top 20 differential KEGG pathway between CK and HM based on q-values.

Transcriptome sequencing of leaves treated with MeJA for 20 d (CK, LM, and HM) produced an average of 6.38 Gb of data per sample, with an average total clean read count of 42.56 M. The average alignment rate of each sample to the genome was 94.45% (Table S1), indicating high sequencing quality. The first principal component (PC1) and the second principal component (PC2) accounted for 87.27% and 5.07% of the variance, respectively, explaining a cumulative total of 92.34% (Figure 4A). This indicates that the data were well explained. The LM and HM group were more dispersed compared to the CK group. This means that MeJA had a notable impact, leading to a broader range of responses. PCA and Pearson’s correlation heatmaps showed that the similarity between HM and CK was lower than that between LM and CK, indicating that high-dose MeJA has a greater impact on gene expression (Figure 4A,B). Therefore, HM and CK were selected for differential analysis, which identified 383 significantly upregulated genes and 506 downregulated genes in the HM group (Figure 4C). GO and KEGG enrichment analyses revealed that differentially expressed genes were mainly enriched in cellular process, catalytic activity, and biological regulation functions, as well as the translation, carbohydrate metabolism, and signal transduction pathways (Figure 4D,F). The top 20 functions and pathways based on q-value are also presented. Overall, differentially expressed genes were primarily enriched in biological process functions and metabolism pathways (Figure 4E,G).

Previous studies identified 57 *TPS* genes in *S. tenuifolia* [12]. The higher overall expression levels of the *TPS* family genes in the LM group compared to the CK and HM groups suggest that moderate concentrations of MeJA may specifically enhance the expression of genes involved in terpene biosynthesis (Table S3). This observation aligns with previous research findings that inducing *S*. *tenuifolia* with the LM group leads to an increase in the density of glandular scales, indicating a direct relationship between MeJA concentration and glandular scale production. The biosynthetic pathway for (+)-menthol in *S. tenuifolia* was documented. This study examined changes in the expression levels of enzymes involved in this pathway following induction with MeJA (Figure 1). The HM group revealed significant intergroup variations in the expression levels of enzymes associated with the (+)-menthol synthesis pathway, suggesting potential instability in reactions induced by high concentrations of MeJA. Notably, the expression levels of the downstream genes *StIPR* and *StPR* in the HM group exceeded those in the CK and LM groups, indicating that high concentrations of MeJA might promote a greater shift toward downstream synthetic processes.

Genome annotation of *S. tenuifolia* indicated that *StTPS5*, *StTPS18*, *StTPS32*, and *StTPS45* are annotated as germacrene D synthase. Notably, the expression level of *StTPS45* was higher (Table S3). Sequence comparison results revealed that *StTPS45* has high similarity to the sesquiterpene synthase of *Lavandula angustifolia*. Thus, *StTPS45* was selected for further gene cloning and heterologous expression.

The sequences of transcription factors (*AtMYC2*, *AaWRKY1*, *AaWRKY9*, *AaERF1*, *AaERF2*) from *Arabidopsis thaliana* and *Artemisia annua* were obtained from NCBI and compared with the *S. tenuifolia* genome using local BLAST analysis to examine their gene expression levels (Table 2). The results indicated that the gene expression level of *Sch000013364* was consistent with the JA-responsive *MYC2*. Alignment with *AaWRKY1* revealed that *Sch000023850*, *Sch000000909*, and *Sch000024381* had multiple matched CDS regions, with *Sch000023850* and *Sch000000909* showing expression levels consistent with JA-responsive *WRKY1*. Other transcription factors did not exhibit a clear pattern. Therefore, it can be inferred that under the LM group, the expression level of *Sch000013364* (partially functioning as *MYC2*) increased, enhancing glandular trichome development and the expression of *TPS* family-related genes in *S. tenuifolia*. Additionally, the increased expression of *Sch000023850* and *Sch000000909* (partially functioning as *WRKY1*) under MeJA induction likely further elevated the expression levels of downstream genes IPR and PR in the (+)-menthol synthesis pathway, thereby increasing the content of these compounds.

### 3.4. Overexpression and Enzyme Assay of the Protein Encoded by the Candidate Gene

In the early stage, *StTPS45* was cloned and prokaryotic expression vector was constructed [12]. The successfully constructed pET32a-*TPS45* recombinant plasmid was introduced into BL21 (DE3) cells for the inducible expression of the target protein. The cells were lysed, and the expressed crude protein was subjected to SDS-PAGE gel electrophoresis. The results (Figure S2) indicated that the cells containing the recombinant plasmid expressed a protein of approximately 72 kDa. This size is consistent with the expected 64.67 kDa of the candidate gene, confirming the successful expression of the target protein. However, compared to the precipitate, the corresponding band in the supernatant was less distinct, suggesting that the TPS45 protein predominantly formed inclusion bodies found in the precipitate.

**Figure 5:**
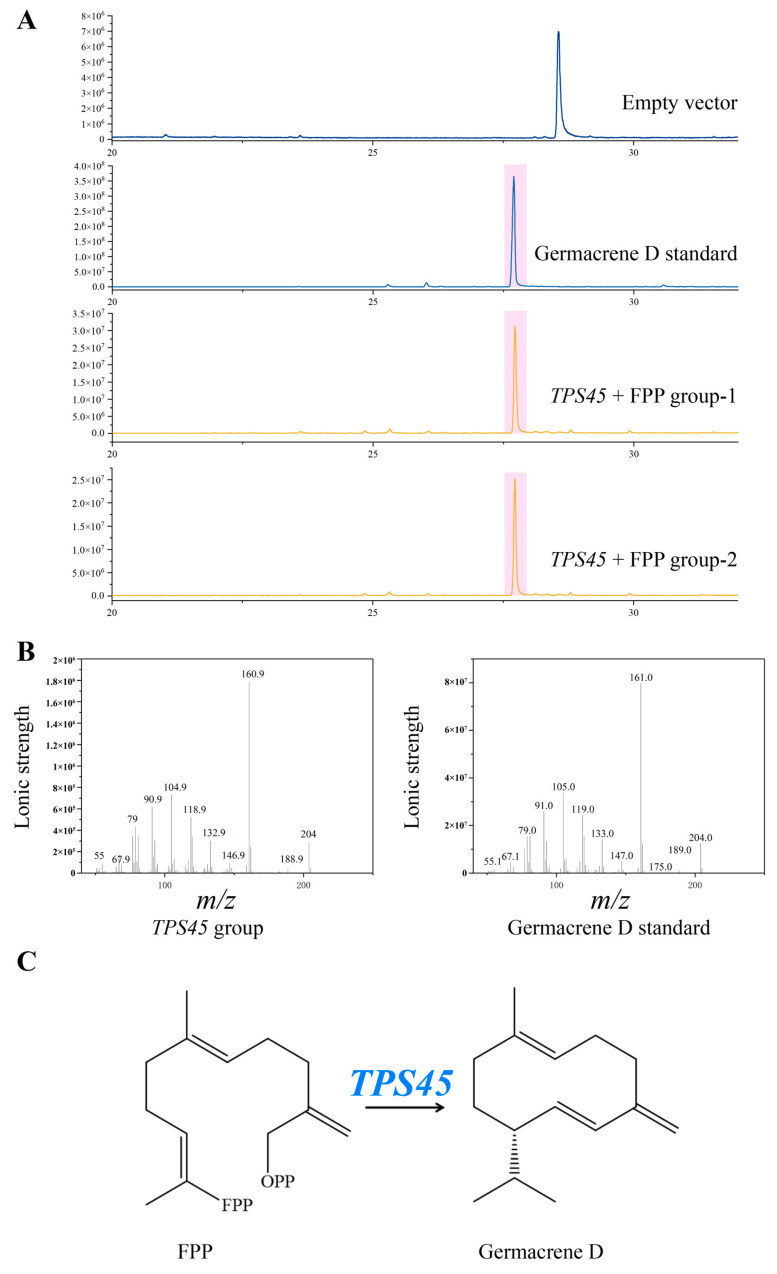
Enzymatic activity and mass spectral analysis of *TPS45* in the synthesis of germacrene D. (**A**) The enzyme activity product of *TPS45* with FPP. (**B**) Mass spectra of the enzymatic product of TPS45 compared with the reference mass spectrum of germacrene D. (**C**) FPP is synthesized into germacrene D under the action of TPS45.

To validate the function of the candidate gene, the crude protein obtained from its prokaryotic expression was reacted with the substrate FPP, and the enzymatic products were analyzed using GC-MS. The experimental results showed a distinct peak at a retention time of 27.789 min for the TPS45 group, in contrast to the negative control with the empty vector, where no corresponding peak was detected (Figure 5A). Additionally, a standard of germacrene D was subjected to GC-MS analysis, revealing a peak at 27.697 min, closely matching the retention time of the TPS45 enzymatic product. Furthermore, comparisons of the mass spectra showed that the ion fragments of the TPS45 enzymatic product were consistent with those of the germacrene D standard (Figure 5B). These results preliminarily confirm that the TPS45 protein can catalyze the formation of germacrene D from FPP, verifying that TPS45 is a germacrene D synthase (Figure 5C).

### 3.5. Physicochemical Property Analysis and Molecular Docking of the Protein Encoded by the Candidate Gene

**Figure 6:**
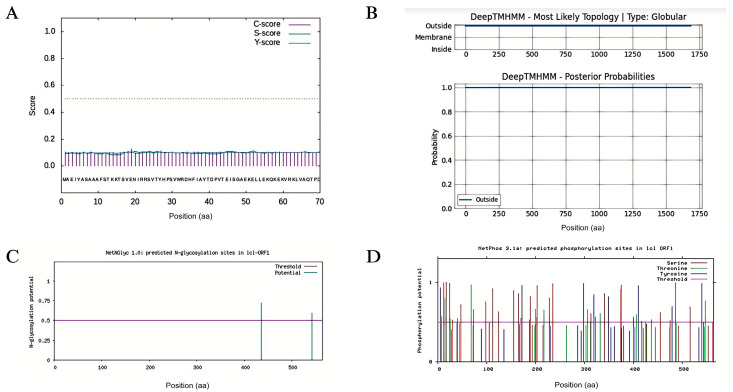
The prediction of *TPS45* protein signal peptide (**A**), protein transmembrane domains (**B**), protein glycosylation sites (**C**), and protein phosphorylation sites (**D**).

Analysis of the TPS45 protein using the Signal P 4.1 Server indicated that the cleavage sites did not exceed the threshold, suggesting that TPS45 does not possess a signal peptide and is a non-secretory protein (Figure 6A). The TMHMM2.0 online tool revealed no transmembrane regions in the protein (Figure 6B). Glycosylation sites predicted by the NetNGlyc 1.0 Server identified potential glycosylation at positions 435 and 542 (Figure 6C). However, as TPS45 lacks a signal peptide, it is unlikely to be glycosylated in vivo, given its probable inaccessibility to N-glycosylation mechanisms. Phosphorylation sites, predicted using NetPhos 3.1 Server, showed that *StTPS45* has 53 potential phosphorylation sites, including 29 serines, 14 threonines, and 10 tyrosines, with a phosphorylation potential greater than 0.5. These sites include binding sites for specific protein kinases such as PKC, PKA, CKII, p38 MAPK, and unsp (Figure 6D).

**Figure 7:**
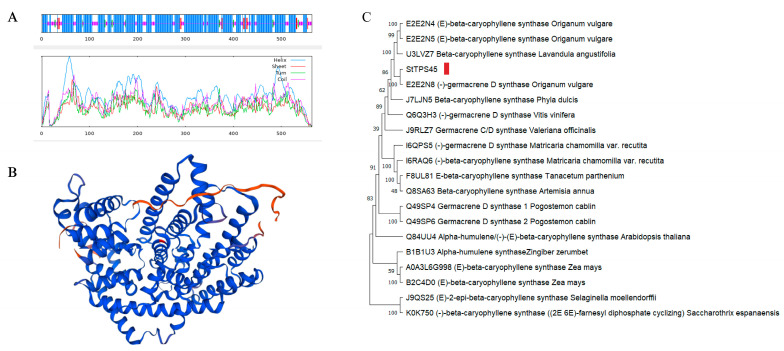
The prediction of TPS45 protein secondary structure (**A**), tertiary structure (**B**), and phylogenetic analysis (the red rectangle marks the location information of StTPS45) (**C**).

Secondary structure analysis of TPS45 protein using SOPMA indicated that α-helices are the predominant structure (70.57%), followed by random coils (23.05%), extended strands (3.55%), and β-turns (2.84%) (Figure 7A). To enhance the credibility of the SWISS-MODEL 3D structural model, a Ramachandran Plot assessed the predicted protein structure, with 93.9% of amino acid residues located in favored regions, validating the TPS45 protein model’s rationality (Figure 7B). The phylogenetic tree shows that the TPS45 sequence is on the same branch as the germacrene D synthase sequence of *Origanum vulgare*, indicating the closest relationship. This further verifies the enzymatic activity of TPS45 (Figure 7C).

**Figure 8:**
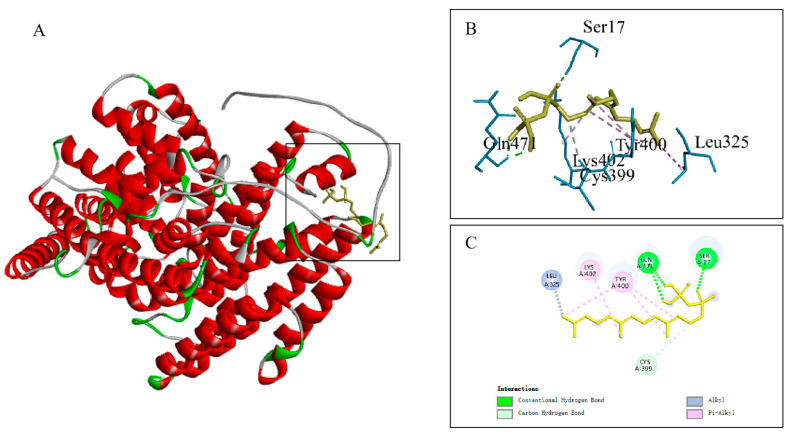
Docking diagram of TPS45 protein with the FPP molecule. The 3D structure of the TPS45 encoded protein and its interaction with FPP (**A**), details of the interaction between the TPS45 encoded protein and FPP (**B**), interaction model of the TPS45 encoded protein with FPP (**C**).

Molecular docking predictions performed between TPS45 protein and the FPP ligand showed a binding energy of −3.51 kcal/mol, indicating binding activity as the binding energy is less than 0 kcal/mol. Additionally, interaction analyses revealed that six amino acids in TPS45 interact with FPP, forming hydrogen bonds at SER-17 and GLN-471 (Figure 8).

## 4. Discussion

This study reveals the impact of MeJA on the volatile oil composition and biosynthetic gene expression in *S. tenuifolia*, highlighting the crucial role of the *StTPS45* gene in terpenoid biosynthesis. To ensure consistency in genetic background, physiological state, and experimental conditions, clonal adventitious shoots were used, confirming that this method had no significant effect on the volatile components of *S. tenuifolia*. Treatment with different concentrations of MeJA resulted in significant changes in the content of major monoterpenes. Transcriptome analysis showed significant changes in gene expression profiles following MeJA treatment, with most *TPS* genes upregulated, particularly under the LM group. Based on gene annotation and expression levels, TPS45 was identified as a functional germacrene D synthase. The *StTPS45* gene was cloned and expressed in *Escherichia coli*, and its function was validated through enzyme activity assays. Molecular docking and structural prediction further revealed specific interactions between TPS45 and its substrate, FPP, consistent with its role in the terpenoid synthesis pathway.

Jasmonate compounds (JAs) are important signaling molecules in plants, playing key roles in various stress responses and developmental processes. Studies have shown that MeJA primarily regulates the biosynthesis, accumulation, and biotechnological applications of secondary metabolites. Current research focuses on gene and promoter cloning, transcription factor regulation, and cell- and tissue-specific analyses [36]. Plant trichomes are often rich in secondary metabolites, and regulating trichome development can significantly increase the content of these compounds, achieving the desired product levels or improved plant phenotypes. MeJA has a significant effect on the development of glandular trichomes and the accumulation of their contents. For example, in mint, MeJA treatment significantly increases glandular trichome density [37]; in thyme, glandular trichomes are known to be the structures for thymol and carvacrol biosynthesis and accumulation. MeJA promotes the high expression of late biosynthetic genes such as *TvTPS1*, *CYP71D178*, and *CYP71D180* in glandular trichomes, resulting in increased thymol production [38]. These phenomena are similar to the increased expression of downstream synthesis genes IPR and PR in *S. tenuifolia* observed in this study following MeJA treatment.

The enhancement in terpenoid production in *S. tenuifolia* by MeJA warrants further investigation. KEGG analysis revealed significant enrichment in signaling pathways, suggesting that MeJA may regulate key transcription factors (such as *MYC2*, *WRKY*, *AP2/ERF*, and *bHLH* families) within these pathways, thereby influencing terpenoid biosynthesis. *MYC2* is a central regulator in the MeJA signaling pathway, controlling the expression of many secondary-metabolism-related genes, including *TPS* genes. MeJA signaling leads to the degradation of JAZ repressor proteins, releasing *MYC2* to activate downstream gene expression [19]. The WRKY transcription factor family plays a critical role in plant stress responses and secondary metabolism. Studies have shown that *WRKY* transcription factors *AaWRKY1* and *AaWRKY9* are regulated by JA, and their overexpression significantly increases artemisinin and dihydroartemisinic acid levels, acting as positive regulators in the artemisinin biosynthesis pathway [39,40]. Additionally, numerous other *WRKY* family transcription factors have been identified as being associated with the synthesis of terpenoids such as artemisinin [41,42]. *AP2/ERF* transcription factors interact with ethylene and jasmonate signaling pathways. Specific *AP2/ERF* transcription factors are activated by JA signaling, regulating secondary metabolism genes and influencing terpenoid synthesis. The *bHLH* family member *AabHLH113* participates in JA signaling by directly binding to the promoters of artemisinin biosynthesis genes *DBR2* and *ALDH1*, positively regulating artemisinin production. The *AP2/ERF* (*AaERF1* and *AaERF2*) and *bHLH* (*AabHLH113*) family transcription factors also play important roles in JA signal regulation [43,44]. According to the results, it can be inferred that under the LM group, the expression level of *Sch000013364* (partially functioning as *MYC2*) increased, enhancing glandular trichome development and the expression of *TPS* family-related genes in *S. tenuifolia*. Additionally, the increased expression of *Sch000023850* and *Sch000000909* (partially functioning as *WRKY1*) under MeJA induction likely further elevated the expression levels of downstream genes IPR and PR in the (+)-menthol synthesis pathway, thereby increasing the content of these compounds.

The selected *TPS45* gene from *S. tenuifolia* was cloned and sequenced, and then ligated into different pET series vectors (such as pET-21a and pET-32a) to construct prokaryotic expression vectors. However, when these vectors were introduced into the BL21 (DE3) expression strain and induced with IPTG, the target TPS45 protein was not significantly expressed. Despite optimizing induction temperature, time, and IPTG concentration, the expression was still unsuccessful. It was hypothesized that the TPS45 protein might contain rare codons [45]. Therefore, the Rosetta (DE3) strain, which carries the pRARE plasmid encoding tRNAs for rare codons (including those for arginine, isoleucine, leucine, proline, and glycine), was used to promote high-frequency expression of genes with rare codons [46]. However, this also did not yield satisfactory results. This suggests a significant codon usage bias between the *TPS45* gene from *S. tenuifolia* and the optimal codon usage in *E. coli*. Consequently, codon optimization of *StTPS45* was performed to improve translation efficiency and enhance TPS45 protein expression in *E. coli c* [47,48,49]. Additionally, using FPP as a substrate in reactions with the TPS45 protein did not produce new products, indicating that no side reactions occurred.

The study of the physicochemical properties and structure of the TPS45 protein in *S. tenuifolia* is crucial for understanding its mechanism of action. The TPS45 protein is a non-secretory protein without transmembrane domains and is primarily composed of α-helices. Its sequence is most closely related to germacrene D synthase in oregano. Molecular docking predictions indicated that the TPS45 protein forms hydrogen bonds with the FPP ligand at SER17 and GLN471, providing direction for further research. Currently, site-directed mutagenesis and fragment swapping techniques are widely used in the study of sesquiterpene synthases, particularly regarding the conserved motifs DDXXD and NSE/DTE [50], and the active site amino acids [51,52,53,54]. The specific roles of active site amino acids in different sesquiterpene synthases vary during the catalytic process. Compared to wild-type sesquiterpene synthases, mutant enzymes may affect product specificity and diversity and alter catalytic efficiency [55]. Investigating site-directed mutations at SER17 and GLN471 in the TPS45 protein and validating their functions can further explore product specificity and catalytic mechanisms.

## 5. Conclusions

This study systematically revealed the impact of MeJA on the volatile oil composition and biosynthetic gene expression in *S. tenuifolia*, highlighting the crucial role of the *TPS45* gene in terpenoid biosynthesis. These findings provide a theoretical basis and potential genetic engineering pathways for optimizing the production of valuable terpenoid compounds in *S. tenuifolia* and other related medicinal plants. Future research could further verify the roles of relevant transcription factors in the MeJA signaling pathway and elucidate their mechanisms in regulating secondary metabolic pathways.

## Figures and Tables

**Table 1:** Identification of the main components of volatile oils in tissue-cultured adventitious bud clusters of *Schizonepeta tenuifolia*.

No.	Compound	Chemical Formula	CAS	RT/min	Relative Content/%
1	*β*-Myrcene	C_10_H_16_	123-35-3	6.090	0.691
2	*d*-Limonene	C_10_H_16_	89-27-5	6.784	4.327
3	*γ*-Terpinene	C_10_H_16_	99-85-4	7.461	0.184
4	1-octen-3-yl-acetate	C_10_H_18_O_2_	2442-10-6	8.869	0.313
5	Trans-p-mentha-2,8-dien-1-ol	C_10_H_16_O	52154-82-2	9.190	0.303
6	Cis-p-mentha-2,8-dien-1-ol	C_10_H_16_O	22771-44-4	9.678	0.213
7	(−)-Carveol	C_10_H_16_O	99-48-9	9.854	0.183
8	(+)-Menthone	C_10_H_18_O	3391-87-5	10.265	2.268
9	(+)-Menthofuran	C_10_H_14_O	17957-94-7	10.685	0.842
10	(−)-Isopulegone	C_10_H_16_O	29606-79-9	11.190	3.737
11	(−)-Verbenone	C_10_H_14_O	1196-01-6	12.956	0.231
12	(−)-Pulegone	C_10_H_16_O	89-82-7	14.724	84.215
13	Piperitenone	C_10_H_14_O	491-09-8	23.108	0.557
14	*β*-Caryophyllene	C_15_H_24_	87-44-5	27.264	1.152
15	Germacrene D	C_15_H_24_	23986-74-5	28.802	0.161
16	*β*-Copaene	C_15_H_24_	3856-25-5	29.806	0.611
17	Cycloheptasiloxane, tetradecamethyl-	C_14_H_42_O_7_Si_7_	107-50-6	31.024	0.241

**Table 2:** The identification of transcription factors related to the JA pathway in *Schizonepeta tenuifolia* was achieved by performing a local BLAST search against the transcription factors from *Arabidopsis thaliana* and *Artemisia annua*.

Transcription Factor	Name	E Value	Grade	Hit Start	Hit End	Gene	CK (FPKM)	LM (FPKM)	HM (FPKM)
*AtMYC2*	HiC_scaffold_6	4.04 × 10^−38^	41.6%	3,066,989	3,067,216	*Sch000027144*	15.22 ± 3.76	11.95 ± 2.46	13.31 ± 6.17
	HiC_scaffold_6	8.90 × 10^−34^	41.2%	3,066,650	3,066,855	*Sch000027144*	15.22 ± 3.76	11.95 ± 2.46	13.31 ± 6.17
	HiC_scaffold_3	2.55 × 10^−34^	41.0%	14,757,041	14,757,259	*Sch000015079*	40.05 ± 4.31	39.08 ± 9.47	30.44 ± 3.88
	HiC_scaffold_3	1.24 × 10^−25^	40.5%	55,950,077	55,949,895	*Sch000013929*	11.11 ± 3.87	7.92 ± 1.8	6.31 ± 0.21
	HiC_scaffold_3	2.09 × 10^−35^	40.1%	14,756,591	14,756,862	*Sch000015079*	40.05 ± 4.31	39.08 ± 9.47	30.44 ± 3.88
	HiC_scaffold_3	5.26 × 10^−24^	39.9%	78,128,356	78,128,177	*Sch000013364*	14.16 ± 1.58	17.29 ± 3.02	13.94 ± 0.43
*AaWRKY1*	HiC_scaffold_508	1.17 × 10^−35^	55.6%	15,781	15,953	*Sch000023850*	14.11 ± 6.54	14.77 ± 5.81	20.81 ± 5.47
	HiC_scaffold_1	1.17 × 10^−35^	55.6%	6,624,000	6,623,828	*Sch000000909*	14.03 ± 6.35	14.63 ± 5.66	20.52 ± 5.31
	HiC_scaffold_4	5.67 × 10^−27^	52.5%	94,706,064	94,706,195	*Sch000016923*	11.59 ± 6.83	10.23 ± 11.14	4.59 ± 4.15
	HiC_scaffold_6	1.25 × 10^−22^	52.3%	77,347,190	77,347,360	*Sch000024381*	2.31 ± 0.81	0.25 ± 0.21	2.53 ± 2.6
	HiC_scaffold_508	8.42 × 10^−25^	52.0%	14,408	14,534	*Sch000023850*	14.11 ± 6.54	14.77 ± 5.81	20.81 ± 5.47
	HiC_scaffold_1	8.42 × 10^−25^	52.0%	6,625,373	6,625,247	*Sch000000909*	14.03 ± 6.35	14.63 ± 5.66	20.52 ± 5.31
	HiC_scaffold_6	1.25 × 10^−22^	51.5%	77,346,493	77,346,632	*Sch000024381*	2.31 ± 0.81	0.25 ± 0.21	2.53 ± 2.6
*AaWRKY9*	HiC_scaffold_6	3.59 × 10^−22^	43.4%	86,739,994	86,740,109	*Sch000027590*	3.02 ± 2.44	1.72 ± 1.12	2.36 ± 1.39
*AaERF1*	HiC_scaffold_6	7.96 × 10^−27^	51.8%	9,096,280	9,096,039	*Sch000027664*	12.24 ± 13.74	15.62 ± 9.39	9.69 ± 6.7
*AaERF2*	HiC_scaffold_4	3.29 × 10^−24^	52.1%	41,015,067	41,015,255	*Sch000018564*	234.55 ± 190.45	148.59 ± 49.31	95.03 ± 9.47

## Data Availability

Data are contained within this article.

## Supplementary Materials

The following supporting information can be downloaded at: https://www.mdpi.com/article/10.3390/plants13141920/s1, Figure S1: Gene optimization of the TPS45 DNA sequence; Figure S2: SDS-PAGE gel electrophoresis image of TPS45 protein expressed in prokaryotic cells; Table S1: Reference genome comparison results; Table S2: Reagent composition and setup for Schizonepeta tenuifolia TPS45 substrate feeding assay; Table S3: The expression levels of genes in the Schizonepeta tenuifolia TPS family.
