# Supplementary material for: Impact of Methyl Jasmonate on Terpenoid Biosynthesis and Functional Analysis of Sesquiterpene Synthesis Genes in Schizonepeta tenuifolia
Source: Plants (Basel). 2024 Jul 12;13(14):1920. doi: 10.3390/plants13141920 (PMC11280979; doi:10.3390/plants13141920)
Supplement: Supplementary file 1 [file plants-13-01920-s001.zip › plants-3083982-supplementary.pdf]

Impact of Methyl Jasmonate on Terpenoid Biosynthesis and Functional Analysis of Sesquiterpene Synthesis Genes in *Schizonepeta tenuifolia*

Dishuai Li[1], Congling Jia[1], Guyin Lin[1], Jingjie Dang[1], Chanchan Liu[1, 2, 3, *], Qinan Wu[1, 2, 3, *]

1 Jiangsu Collaborative Innovation Center of Chinese Medicinal Resources Industrialization, Nanjing University of Chinese Medicine, Nanjing 210023, China

2 State Key Laboratory on Technologies for Chinese Medicine Pharmaceutical Process Control and Intelligent Manufacture, Nanjing University of Chinese Medicine, Nanjing 210023, China

3 School of Pharmacy, Nanjing University of Chinese Medicine, Nanjing 210023, China; 20210651@njucm.edu.cn (D.L.); jiacongling@njucm.edu.cn (C.J.); lgy_grace@sina.com (G.L.); jingjiedang@njucm.edu.cn (J.D.)

* Correspondence: liuchanchan@njucm.edu.cn (C.L.); wuqn@njucm.edu.cn (Q.W.)

Original ATGGCCGAAATCTATGCATCGGCTGCTGCCTTCTCCACAAAGAAAACAAGTGTAGAGAATATTCGCCGATCGGTAACGTATCATCCCAGC
Optimized ATGGCTGAAATCTACGCGAGCGCGGCGGCGTTCAGCACCAAAAAGACCTCTGTTGAAAACATCCGTCGCTCTGTTACCTACCATCCGTCT

Original GTTTGGAGAGATCATTTTATTGCATATACTGACCCCGTTACGGAAATCAGTGGTGCCGAGAAGGAACTACTCGAAAAGCAAAAGGAAAAG
Optimized GTTTGGCGTGACCACTTCATTGCCTACACCGATCCGGTTACCGAAATCAGCGGCGCTGAAAAAGAACTGCTGGAAAAACAGAAAGAAAAA

Original GTAAGGAAGTTGGTAGCTCAAACTCCAGATGATTCAACGCTCAAGATCGAGCTCATCGATGCAATCCAACGTCTAGGTATTGGCTATCAT
Optimized GTGCGTAAACTGGTTGCGCAGACCCCGGACGATAGCACCCTGAAAATCGAACTGATCGATGCGATCCAGCGTCTGGGCATCGGCTACCAC

Original TTTGAAAAAGAAATCAAAGAATCCTTACGACACATTCACGACAGTTGCCAAATACAAATTGCAAGCAAAGACGACGATGATGATGATGTT
Optimized TTCGAAAAAGAAATCAAAGAATCTCTGCGTCACATCCATGATAGCTGCCAGATTCAGATCGCAAGCAAAGACGATGATGACGATGACGTG

Original GGTGTTGTTTCTCTTCGCTTTCGTTTGCTAAGACAACAAGGTTACCGCGTCCCATGTGATGTGTTCAATAAATTGATAGACGACAAGGGC
Optimized GGCGTTGTTTCCCTGCGTTTTCGTCTGCTGCGTCAGCAGGGCTACCGTGTTCCGTGTGATGTGTTCAACAAACTGATCGACGATAAAGGT

Original AATTTTAAAGAGTCTCTAATAAACAATGTTGAAGGAATGCTAAGCTTATACGAGGCTTCAAATTATGAAATAAATGGAGAGGAAATTCTT
Optimized AACTTCAAAGAATCCCTGATTAACAACGTTGAAGGTATGCTGAGCCTGTACGAAGCTAGCAACTACGAAATTAACGGTGAAGAAATCCTG

Original GACAAAGCCTTAGAATTTTCTTCTTCTCATCTCGAATCTTTAATGCCTCAAACTACTACTTCTCTCTCGAGGCGAATCAAAGAAGCTTTG
Optimized GATAAAGCACTGGAATTCAGCAGCAGCCACCTGGAAAGCCTGATGCCGCAGACCACCACCTCCCTGAGCCGTCGTATCAAAGAAGCTCTG

Original GAGATGCCAATTAGCAAGACTTTGATGAGATTGGGGCGAGGAAATTCATATCTTTGTACCAAGAAGATGAGTCGCATAATGAAATATTA
Optimized GAAATGCCGATCTCTAAAACCTTGATGCGTCTGGGCGCGCGTAAATTTATTAGCCTGTACCAGGAAGATGAATCCCACAACGAAATCCTG

Original TTGGATTTTGCAAAATTGGACTTCAATATAATTCAGAAGATGCACCAAAGAGAGCTACACCATATTACAAGGTGGTGGAAGGATTTAGAT
Optimized CTGGATTTCGCTAAACTGGATTTCAACATCATCCAGAAAATGCATCAGCGTGAACTGCACCATATCACCCGTTGGTGGAAAGATCTGGAT

Original TTTGCAAATAAACTACCTTTTGCAAGAGATAGAGTGGTGGAGTGCTACTTTTGGATTTTGGAGTCTATTTTGAGCCAAAATACGAAATT
Optimized TTCGCTAACAAACTGCCGTTTGCGCGTGATCGTGTTGTTGAATGCTACTTCTGGATTCTGGGCGTTTACTTCGAACCGAAATACGAAATT

Original GCAAGAACATTTCTAACCAAAGTCATAGCTGTGACATCCATCCTTGATGATATTTATGATGTTTATGGGAATTTAGATGAACTTGGAGGT
Optimized GCGCGTACCTTCCTGACCAAAGTGATCGCAGTTACCTCCATTCTGGATGACATCTACGACGTTTACGGTAACCTGGATGAACTGGGCGGC

Original TTCACCGATGCTATTGAAAGATGGGATATTAGTGCTGTTGATGAATTGCCACCATACATGAGAATATGTTACGTAGCTCTTTTAGGGTT
Optimized TTCACCGATGCCATCGAACGCTGGGACATCAGCGCAGTTGATGAACTGCCGCCGTACATGCGTATCTGCTACGTTGCGCTGCTGGGCGTG

Original TATGCCGAAATGGAAGATGAAATGATAAAACAAGGTGAATCATCATATCGCCTTCAATATGCAAAACAAGAGATGATAAAATTGGTGGCG
Optimized TACGCTGAAATGGAAGATGAAATGATCAAACAGGGCGAATCTAGCTATCGCCTGCAGTACGCGAAACAGGAAATGATCAAACTGGTTGCG

Original GCATATATGGAAGAGGCAGAATGGTGTTACAACAAGTATATTCCGACAATGGAGGAGTATATGAAATTAGCACTTGTATCTGGCGCTTAT
Optimized GCGTACATGGAAGAAGCGGAATGGTGCTACAATAAATACATCCCGACCATGGAAGAATACATGAAACTGGCGCTGGTGTCCGGTGCGTAC

Original ATGATGCTAGCAACAACTTCTTTAGTCGGAATGAGAGATCATAATATTACACAACAAGATTTTGATTGGATTACAAACCAACCACCCATT
Optimized ATGATGCTGGCAACCACCTCTCTGGTTGGTATGCGTGACCACAACATCACCCAGCAGGATTTCGACTGGATCACCAACCAGCCGCCGATT

Original TTACAAGCCGCGTCGGTTATTTGTAGATTAATGGACGACATGGTAGGACATGGGATTGAGCAAAAAATTACAAGCGTGGATTGTTACATG
Optimized CTGCAGGCGGCGAGCGTTATCTGCCGCCTGATGGATGACATGGTGGGCCACGGTATCGAACAGAAAATTACCAGCGTGGACTGCTACATG

Original AAGGAAAATCGGTGCTCAAAGACGGAAGCTTGTAGTGAATTCTGGAAGCGAGTGAAGAAAGCGTGGAAGGATATGAATGAGGAATGCCTG
Optimized AAAGAAAACCGTTGCAGCAAAACCGAAGCGTGTAGCGAATTCTGGAAACGTGTTAAAAAAGCGTGGAAAGATATGAACGAAGAATGTCTG

Original GAGCCAAGAGCAGCATCTATGGCGATTCTTATGCGCGTTGTTAATCTTGCTCGCGTCATAAATTTACTGTACGTTGGTGAAGATGGATAT
Optimized GAACCGCGTGCGGCAAGCATGGCGATCCTGATGCGTGTGGTTAACCTGGCGCGTGTTATCAACCTGCTGTACGTGGGTGAAGATGGTTAT

Original GGTAATTCCACTACCAACACCAAAGATTTAATTAAATCTGTGCTCGTGGATCCCCTACACTGTTCGAATTGTTAA
Optimized GGTAACAGCACCACCAACACCAAAGACCTGATCAAAAGCGTTCTGGTTGATCCGCTGCACTGTAGCAACTGCTAA

Figure S1. Gene optimization of the *TPS45* DNA sequence.

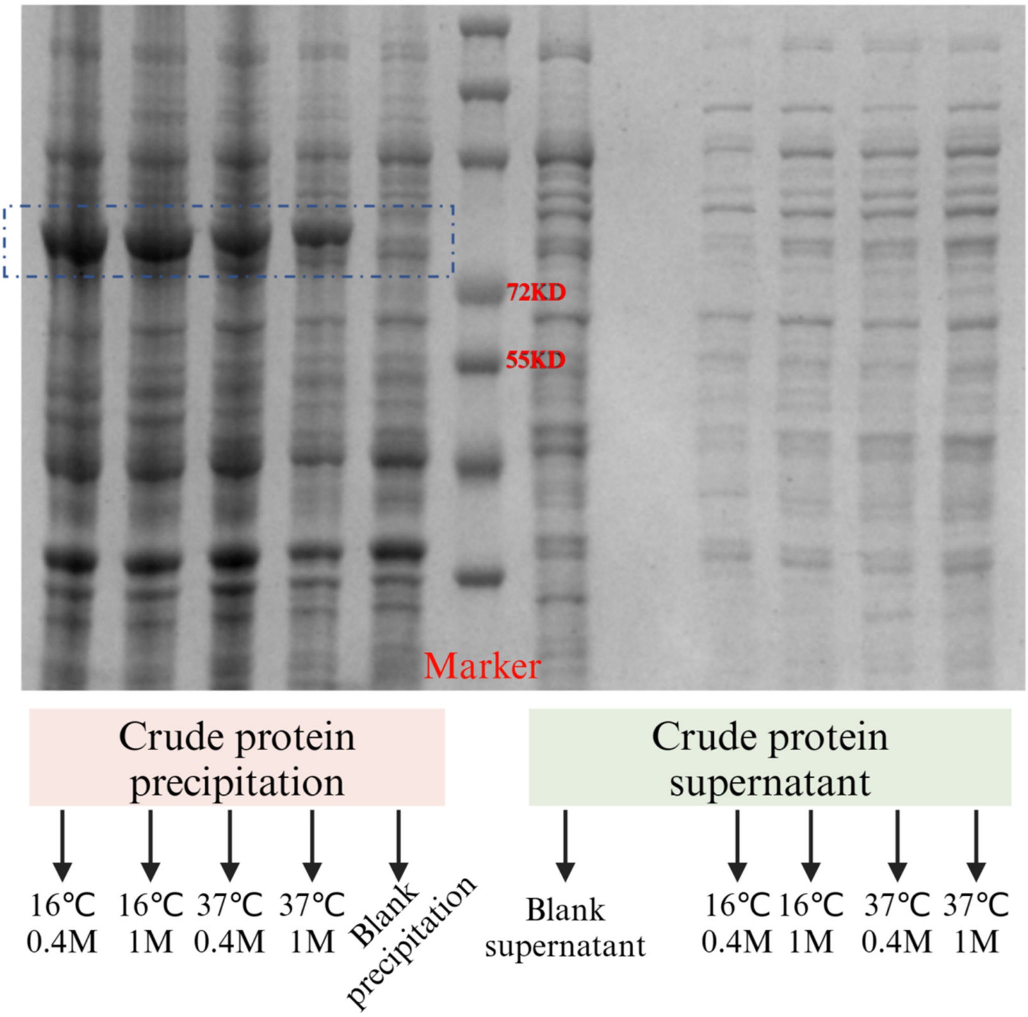

Figure S2. SDS-PAGE gel electrophoresis image of *TPS45* protein expressed in prokaryotic cells.

Note: The marker range is 10-170 kDa, and the boxed region indicates the target protein *TPS45*.

Table S1. Reference genome comparison results.

Sample name	Total Clean Bases (Gb)	Total clean read (M)	Total mapping genome ratio (%)
CK_1	6.38	42.52	95.01
CK_2	6.37	42.48	95.04
CK_3	6.51	43.37	94.78
HM_1	6.36	42.42	94.59
HM_2	6.37	42.44	94.65
HM_3	6.36	42.38	93.12
LM_1	6.48	43.23	94.83
LM_2	6.33	42.18	94.91
LM_3	6.30	42.00	93.12

Table S2. Reagent composition and setup for *Schizonepeta tenuifolia TPS45* substrate feeding assay.

No.	Reagent	Dosage
1	1 M Tri-HCl（pH =7）	50 μL
2	1 M $MgCl_2$	5 μL
3	100 m DTT	5 μL
4	FPP	10 μL
5	Crude enzyme solution	430 μL

Table S3. The expression levels of genes in the Schizonepeta tenuifolia TPS family.

No.	Gene name	Scaffold	CK (FPKM)	LM (FPKM)	HM (FPKM)
StTPS1	*Sch000000787*	HiC_scaffold__1	23.82±7.87	15.97±14.61	5.55±4.95
StTPS2	*Sch000000817*	HiC_scaffold__1	2.67±0.54	2.39±0.19	1.92±0.26
StTPS3	*Sch000001900*	HiC_scaffold__1	0.02±0.04	0.00±0.00	0.09±0.07
StTPS4	*Sch000002290*	HiC_scaffold__1	6.44±5.44	4.00±0.30	0.93±0.26
StTPS5	*Sch000004023*	HiC_scaffold__1	0.29±0.47	0.11±0.20	0.00±0.00
StTPS6	*Sch000004272*	HiC_scaffold__1	0.59±0.57	0.23±0.08	0.13±0.05
StTPS7	*Sch000004380*	HiC_scaffold__1	0.45±0.28	0.33±0.05	0.68±0.20
StTPS8	*Sch000004666*	HiC_scaffold__102	0.03±0.03	0.08±0.09	0.08±0.03
StTPS9	*Sch000004930*	HiC_scaffold__13	2.41±0.53	1.69±0.89	2.42±0.94
StTPS10	*Sch000005332*	HiC_scaffold_2	1.62±0.78	1.07±0.60	0.55±0.18
StTPS11	*Sch000007539*	HiC_scaffold_2	2.74±1.25	2.05±1.05	3.13±2.89
StTPS12	*Sch000009262*	HiC_scaffold_2	0.00±0.00	0.00±0.00	0.00±0.00
StTPS13	*Sch000009837*	HiC_scaffold_2	0.21±0.27	0.10±0.05	0.10±0.12
StTPS14	*Sch000010201*	HiC_scaffold_25	0.00±0.01	0.00±0.00	0.00±0.00
StTPS15	*Sch000010232*	HiC_scaffold_254	0.00±0.00	0.00±0.00	0.00±0.00
StTPS16	*Sch000011302*	HiC_scaffold_3	0.12±0.08	0.03±0.02	0.00±0.00

StTPS17	*Sch000011609*	HiC_scaffold_3	0.57±0.73	0.42±0.26	0.50±0.41
StTPS18	*Sch000011615*	HiC_scaffold_3	0.01±0.01	0.00±0.00	0.00±0.00
StTPS19	*Sch000012975*	HiC_scaffold_3	16.48±8.03	25.58±12.98	11.18±2.42
StTPS20	*Sch000013152*	HiC_scaffold_3	8.25±3.53	5.41±3.03	7.00±5.59
StTPS21	*Sch000013374*	HiC_scaffold_3	0.02±0.03	0.00±0.00	0.00±0.00
StTPS22	*Sch000013781*	HiC_scaffold_3	0.00±0.00	0.02±0.03	0.00±0.00
StTPS23	*Sch000014410*	HiC_scaffold_3	0.19±0.05	0.27±0.12	0.25±0.13
StTPS24	*Sch000015054*	HiC_scaffold_3	0.15±0.20	0.35±0.57	0.03±0.04
StTPS25	*Sch000015185*	HiC_scaffold_3	6.05±2.45	5.37±3.18	5.59±4.66
StTPS26	*Sch000015357*	HiC_scaffold_3	0.61±0.32	5.81±5.60	12.97±13.54
StTPS27	*Sch000015681*	HiC_scaffold_3	10.10±2.81	8.86±5.13	5.53±3.62
StTPS28	*Sch000018518*	HiC_scaffold_4	2.09±1.84	7.46±8.56	1.91±1.09
StTPS29	*Sch000019433*	HiC_scaffold_4	5.61±9.00	0.73±1.25	0.30±0.36
StTPS30	*Sch000020959*	HiC_scaffold_5	1.40±0.52	0.26±0.15	0.01±0.02
StTPS31	*Sch000021024*	HiC_scaffold_5	0.00±0.00	0.00±0.00	0.00±0.00
StTPS32	*Sch000021194*	HiC_scaffold_5	1.45±0.96	0.30±0.13	1.93±2.68
StTPS33	*Sch000021207*	HiC_scaffold_5	0.06±0.11	0.00±0.00	0.01±0.02
StTPS34	*Sch000021894*	HiC_scaffold_5	0.00±0.00	0.00±0.00	0.00±0.00
StTPS35	*Sch000021999*	HiC_scaffold_5	40.97±36.33	57.07±51.02	19.24±13.90
StTPS36	*Sch000022152*	HiC_scaffold_5	0.85±0.59	0.01±0.02	0.26±0.19
StTPS37	*Sch000022335*	HiC_scaffold_5	0.10±0.04	0.42±0.42	0.03±0.05
StTPS38	*Sch000022498*	HiC_scaffold_5	1.43±0.63	1.91±0.79	0.95±0.12
StTPS39	*Sch000022539*	HiC_scaffold_5	37.53±33.67	44.53±29.69	15.95±14.24
StTPS40	*Sch000022645*	HiC_scaffold_5	92.13±2.80	127.48±68.07	35.71±24.21
StTPS41	*Sch000022844*	HiC_scaffold_5	0.00±0.00	0.02±0.04	0.01±0.02
StTPS42	*Sch000022938*	HiC_scaffold_5	0.99±0.77	5.36±8.76	0.11±0.12
StTPS43	*Sch000023444*	HiC_scaffold_5	17.28±13.74	22.38±12.95	7.91±6.37
StTPS44	*Sch000023607*	HiC_scaffold_5	45.98±51.91	49.86±44.97	12.21±7.74
StTPS45	*Sch000024754*	HiC_scaffold_6	17.71±4.84	7.09±0.53	9.43±3.85
StTPS46	*Sch000025042*	HiC_scaffold_6	1.24±0.64	0.93±0.86	0.91±0.76
StTPS47	*Sch000025070*	HiC_scaffold_6	0.51±0.46	0.29±0.17	8.27±7.33
StTPS48	*Sch000025126*	HiC_scaffold_6	0.07±0.12	0.00±0.00	0.00±0.00
StTPS49	*Sch000026106*	HiC_scaffold_6	40.94±28.40	4.48±2.40	37.84±56.85
StTPS50	*Sch000026573*	HiC_scaffold_6	0.03±0.05	0.00±0.00	0.00±0.00
StTPS51	*Sch000026886*	HiC_scaffold_6	0.03±0.05	0.01±0.02	0.02±0.03
StTPS52	*Sch000026966*	HiC_scaffold_6	35.48±24.06	3.59±1.91	34.62±54.30
StTPS53	*Sch000027775*	HiC_scaffold_6	0.02±0.03	0.02±0.03	0.04±0.06
StTPS54	*Sch000028051*	HiC_scaffold_6	0.00±0.00	0.00±0.00	0.00±0.00
StTPS55	*Sch000028090*	HiC_scaffold_6	0.15±0.09	0.12±0.04	0.06±0.02
StTPS56	*Sch000029354*	HiC_scaffold_6	3.65±3.18	18.67±13.75	8.57±10.43
StTPS57	*Sch000029479*	HiC_scaffold_634	0.00±0.00	0.00±0.00	0.00±0.00
